# Neuropsychiatric Symptoms, Gray Matter Microstructural Integrity and Alzheimer’s Disease Pathology in Older Adults with Amnestic Mild Cognitive Impairment and Dementia of Alzheimer’s Type

**DOI:** 10.1002/alz70862_110159

**Published:** 2025-12-23

**Authors:** Quentin Devignes, Patrick J. Pruitt, Kenneth Petscavage, Robert A. Koeppe, Roger L. Albin, Scott J. Peltier, Annalise Rahman‐Filipiak, Bruno Giordani, Benjamin M. Hampstead, Alexandru D. Iordan

**Affiliations:** ^1^ University of Michigan, Ann Arbor, MI USA; ^2^ Michigan Alzheimer's Disease Research Center, Ann Arbor, MI USA; ^3^ VA Ann Arbor Healthcare System, Ann Arbor, MI USA

## Abstract

**Background:**

Neuropsychiatric symptoms (NPS) are increasingly acknowledged as core symptoms of Alzheimer’s disease (AD). Studies have reported significant associations between NPS severity and disruption of salience network brain functional connectivity. However, the pathophysiological and structural correlates of NPS in AD are less clear. This study aimed to examine the relationships of NPS severity with gray matter (GM) microstructural integrity and amyloid and tau burden in older adults with amnestic mild cognitive impairment (aMCI) and mild dementia of Alzheimer’s type (DAT).

**Method:**

We analyzed data from 178 participants (mean age: 72.01±7.01; 43.82% female) with aMCI (66.29%) or DAT (33.71%). For each participant, an informant completed the Neuropsychiatric Inventory Questionnaire (NPIQ) to evaluate NPS severity. Participants underwent PET using [^11^C]‐Pittsburgh compound B (PiB) and [^18^F]AV‐1451 to evaluate amyloid and tau deposition, respectively, and a 3T MRI scan including a diffusion‐weighted sequence. Four voxel‐wise linear regression models evaluated the relationship of NPIQ total score with (a) PiB uptake, (b) [^18^F]AV‐1451 uptake, and two indices derived from neurite orientation dispersion and density imaging to evaluate GM microstructural integrity, namely (c) neurite density index (NDI) and (d) orientation dispersion index (ODI). All analyses controlled for age, sex, and educational level. Multiple comparison correction was applied using *p*‐uncorrected<.001 at the voxel level and family‐wise error *p*‐corrected<.05 at the cluster level.

**Result:**

Higher NPIQ total score was significantly associated with lower ODI in the right insula and right inferior frontal gyrus (*pars opercularis*, *triangularis* and *orbitalis*). There were no significant associations with NDI and PiB and [^18^F]AV‐1451 uptakes. At a more lenient significance threshold (voxel‐level *p*‐uncorrected<.005), higher NPIQ was associated with higher [^18^F]AV‐1451 uptake in the left striatum.

**Conclusion:**

Our findings show that more severe NPS are associated with altered diffusivity in cortical regions canonically associated with the salience network, which may reflect reduced dendritic arborization complexity in these regions. Our results suggest that NPS severity may be more tightly tied to microstructural GM changes than AD pathology as measured via PET. These results support prior functional MRI studies identifying the salience network as the primary network of interest for NPS in those with AD.

